# Supplementary figures and images for: High sorbic acid resistance of Penicillium roqueforti is mediated by the SORBUS gene cluster
Source: PLoS Genet. 2022 Jun 15;18(6):e1010086. doi: 10.1371/journal.pgen.1010086 (PMC9200314; doi:10.1371/journal.pgen.1010086)

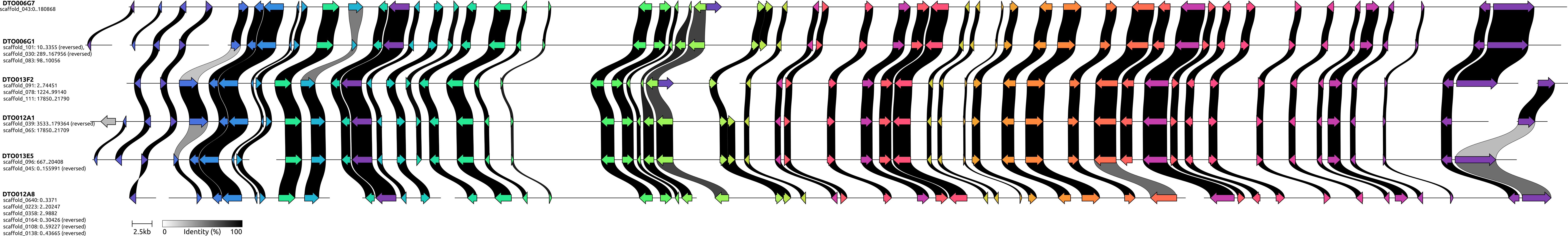

Supplement: S1 Fig — Gene identity (%) is indicated in greyscale and (partial) scaffolds corresponding to scaffold 43 of DTO006G7 are listed in order below the strain names. (TIFF) [file pgen.1010086.s002.tiff]

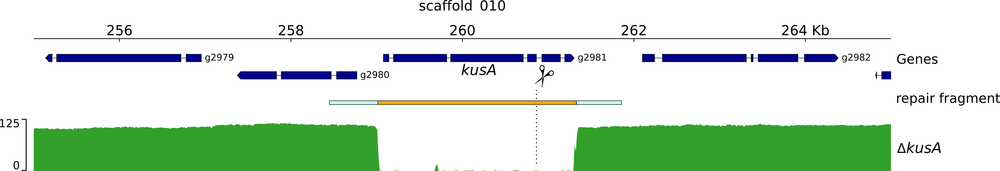

Supplement: S2 Fig — Genes are indicated with blue arrows. The nanopore sequencing read coverage of DTO013F2 ΔkusA is indicated in green. The orange bar indicates the targeted knock-out region, flanked by the 5’ and 3’ ends (in lightblue). Dotted lines and scissors indicate the loci targeted by the sgRNA. (TIFF) [file pgen.1010086.s003.tiff]
